# Mechanisms of Resistance to CAR T-Cells and How to Overcome Them

**DOI:** 10.3390/mps8050108

**Published:** 2025-09-11

**Authors:** Luca Legato, Matteo Bisio, Filippo Fasano, Corrado Benevolo Savelli, Carolina Secreto, Chiara Maria Dellacasa, Barbara Botto, Alessandro Busca, Marco Cerrano, Roberto Freilone, Mattia Novo

**Affiliations:** Division of Hematology and Allogeneic Stem Cell Transplant Unit, A.O.U. Città della Salute e della Scienza di Torino, C.so Bramante 88, 10126 Turin, Italy; mbisio@cittadellasalute.to.it (M.B.); ffasano@cittadellasalute.to.it (F.F.); cbenevolosavelli@cittadellasalute.to.it (C.B.S.); csecreto@cittadellasalute.to.it (C.S.); cdellacasa@cittadellasalute.to.it (C.M.D.); bbotto@cittadellasalute.to.it (B.B.); abusca@cittadellasalute.to.it (A.B.); mcerrano@cittadellasalute.to.it (M.C.); rofreilone@cittadellasalute.to.it (R.F.)

**Keywords:** CAR T-cells, B-cell lymphoma, B-cell acute lymphoblastic leukemia, multiple myeloma, dual targeting, allogeneic CAR T-cells, CAR NK, CAR T-cell resistance

## Abstract

In the last few decades, chimeric antigen receptor (CAR) T-cell therapy has led to a paradigm shift in the treatment of hematological malignancies, including various subtypes of B-cell non-Hodgkin’s lymphoma, B-cell acute lymphoblastic leukemia, and multiple myeloma. However, most patients experience refractoriness to CAR T-cells or relapse after treatment. Many efforts are underway to understand the mechanisms behind CAR T-cell failure, which are mainly related to CAR T-cell dysfunction, tumor-intrinsic resistance, an immunosuppressive tumor microenvironment, manufacturing issues, or patient-related factors. Several strategies are being developed to overcome these resistance mechanisms, including the engineering of more functional allogeneic CAR T-cell products, the targeting of alternative tumor antigens, and combination therapies with other drugs such as checkpoint inhibitors or small molecules to enhance CAR T-cell efficacy. In this review, we will provide an updated overview of the mechanisms of CAR T-cell failure and the therapeutic advances currently under development to address them.

## 1. Introduction

Chimeric antigen receptor (CAR) T-cell therapy represents the most relevant advance for patients with hematological malignancies of the last few decades, since it significantly impacted the treatment paradigm of a wide spectrum of lymphoproliferative disorders. CAR T-cells are autologous or allogeneic T-lymphocytes genetically engineered to express synthetic receptors that redirect specificity against tumor-associated antigens. To date, four anti-CD19 autologous CAR T-cell products—axicabtagene ciloleucel (axi-cel), brexucabtagene autoleucel (brexu-cel), lisocabtagene maraleucel (liso-cel), and tisagenlecleucel (tisa-cel)—are approved for treatment of relapsed or refractory (R/R) B-cell acute lymphoblastic leukemia (B-ALL) and B-cell non-Hodgkin lymphomas (B-NHLs), including large B-cell lymphomas (LBCL), follicular lymphoma (FL), and mantle-cell lymphoma (MCL); in addition, two anti-B-cell maturation antigen (BCMA) constructs—ciltacabtagene autoleucel (cilta-cel) and idecabtagene vicleucel (ide-cel)—are commercially available for multiple myeloma (MM) [1,2,3,4,5,6,7,8,9,10,11]. Moreover, several CAR T-cell products are under development for other hematological malignancies as well as solid tumors [12,13,14,15].

Despite the outstanding efficacy of CAR T-cells, most patients experience progressive disease or subsequently relapse. The resistance to CAR T-cell treatment can be due to different mechanisms: CAR T-cell-dependent issues (manufacturing failure or CAR T-cell dysfunction), tumor-intrinsic factors (antigen escape or tumor microenvironment elements), or CAR T-cell and patient-related determinants (prior antineoplastic treatment exposure or gut microbiome) [16,17].

Therefore, understanding and overcoming the resistance mechanisms of CAR T-cells is one of the most critical goals in hematological research. Many strategies are being investigated to ameliorate CAR T-cell anticancer performance, including the adoption of different targets, the resort to different cell sources, or the combination with other drugs with a potential synergistic effect. This review aims to provide an up-to-date overview of these resistance mechanisms and to illustrate how research is progressing to overcome them, with the ultimate goal of improving the efficacy of CAR T-cell therapies and patient outcomes.

## 2. Mechanisms of CAR T-Cell Resistance

### 2.1. CAR T-Cell Dysfunction

The expansion and persistence of chimeric antigen receptor (CAR) T-cells are critical determinants for achieving complete responses and preventing relapses in hematological malignancies [18,19].

In autologous CAR T-cell therapy, the available T-cell pool used may be sub-optimal for several reasons, including patient age, prior cytotoxic or lymphotoxic therapies, and chronic antigen stimulation.

Adoptive cell transfer of T-cells, which entails ex vivo expansion and reinfusion of antigen-specific T-lymphocytes—including CAR T-cell therapy—has shown improved efficacy and a better toxicity profile when the infused T-cell pool predominantly consists of less differentiated T-cells, such as naïve T-cells (T_N_), stem cell memory T-cells (T_SCM_), and central memory T-cells (T_CM_). This is likely due to their superior expansion capacity, long-term persistence, and potential to differentiate into effector cells compared to more differentiated subsets such as effector memory T-cells (T_EM_) and effector T-cells (T_Eff_) and to dysfunctional subsets including exhausted (T_Ex_), senescent, and anergic T-cells [20,21,22,23].

#### 2.1.1. Memory T-Cells

Naïve T-cells expand and differentiate into effector cells upon antigenic stimulation, a fraction of which evolve into memory T-cells (T_MEM_). T_MEM_ cells can be further classified based on their biological and immunophenotypic characteristics. T_EM_ cells exhibit strong effector functions but limited self-renewal capacity and long-term persistence, and they lack lymph node homing ability. In contrast, T_CM_ cells retain self-renewal and persistence capabilities, preserve lymph node homing, and can differentiate into both T_EM_- and T_Eff_-cells. T_SCM_ cells possess the highest levels of self-renewal and persistence, as well as the ability to differentiate into T_CM_-, T_EM_-, and T_Eff_-cells [20].

A preclinical study in both murine and human models demonstrated that the coexistence of T_MEM_- and T_N_-cells during ex vivo expansion and subsequent in vivo co-transfer can induce premature T_N_ differentiation toward T_EM_- and T_Eff_-cells rather than T_CM_- and T_SCM_-cells, hampering the antitumor efficacy of the resulting product. The phenomenon is dose-dependent (linked to the T_MEM_/T_N_ ratio) and mediated by non-apoptotic Fas/FasL interactions that activate AKT and ribosomal S6 protein (S6)—key regulators of cellular metabolism and differentiation [22]. This study also showed that the T_MEM_/T_N_ ratio was markedly elevated in heavily pre-treated patients with LBCL compared to matched healthy donors, presumably due to prior lymphotoxic treatments—an observation of critical relevance given the widespread clinical use of unfractionated T-cell products in CAR T-cell therapy. Finally, transcriptomic analyses of CAR T-cells derived from patients with chronic lymphocytic leukemia (CLL) revealed that responder-derived CAR T-cells exhibited high expression of memory-associated genes, whereas non-responder-derived CAR T-cells were enriched for genes related to effector differentiation, glycolysis, exhaustion, and apoptosis. Notably, durable remission correlated with a high frequency of T-cells carrying a phenotype consistent with T_SCM_-cells [24].

#### 2.1.2. Dysfunctional T-Cells

Dysfunctional T-cells, including anergic, senescent, and T_Ex_-cells, represent a major barrier to the effectiveness of CAR T-cell therapy.

Anergic T-cells result from suboptimal antigen stimulation and show low proliferative, cytotoxic, and cytokine-secreting capabilities. Conversely, senescent T-cells arise from repeated antigenic stimulation or cellular stress (e.g., reactive oxygen species, chemotherapy) and experience cell cycle arrest due to telomere shortening or DNA damage. These cells have low proliferation, reduced expression of costimulatory receptors (CD27, CD28), and diminished cytotoxic function. However, unlike T_Ex_-cells, they retain high cytokine-secretion capacity and acquire a unique “senescence-associated secretory phenotype”, producing both pro-inflammatory cytokines (IL-2, IL-6, TNF-α, IFN-γ) and immunosuppressive ones (IL-10, TGF-β). In contrast, T_Ex_-cells result from chronic antigen stimulation (e.g., chronic infections or cancer) and are characterized by poor proliferation, high expression of inhibitory receptors (IRs) such as PD-1, CTLA-4, TIM-3, LAG-3, and TIGIT, and reduced cytotoxicity and cytokine secretion [21,25,26]. The IRs act synergistically to induce immune tolerance via two main mechanisms: competition with costimulatory receptors (e.g., CTLA-4 competes with CD28 for binding to CD80/CD86) and transmission of inhibitory signals that suppress TCR-mediated T-cell activation (e.g., PD-1/PD-L1 interaction) [21,25]. The exhausted phenotype is regulated by both intrinsic (transcription factors) and extrinsic (cytokines) factors, resulting from complex T-cell–microenvironment interactions. A recent study using both preclinical models and samples from responders and non-responders enrolled in the pivotal ZUMA-1 trial showed that IL-4 promotes an exhausted-like phenotype in CAR T-cells, suggesting that IL-4 inhibition may enhance therapeutic efficacy [27]. In the JULIET trial, a higher PD-1/PD-L1 interaction score (defined as the percentage of PD-1^+^ cells co-localized with PD-L1^+^ cells) and a higher percentage of LAG3^+^ T-cells were associated with a greater risk of refractory disease and a greater risk of relapse [5].

#### 2.1.3. Age-Related Changes in T-Cell Subsets

A recent study revealed significant age-related changes in T-cell subpopulations of healthy individuals. Specifically, there was a decrease in both the absolute and relative numbers of CD4^+^- and CD8^+^-naïve T-cells, an absolute reduction in CD8^+^ TSCM cells, a relative increase in CD8^+^ CD28^−^ T-cells (indicative of senescent CD8^+^ T-cells), and an increase in both absolute and relative numbers of CD4^+^ CD28^−^ T-cells (indicative of senescent CD4^+^ T-cells) [28]. These immunological shifts reflect the process of immunosenescence and might theoretically affect CAR T-cell manufacturing and function. However, clinical evidence to date does not demonstrate consistent differences in efficacy between younger and older patients, with comparable overall remission rates (ORR) and complete response (CR) rates, progression-free survival (PFS), and overall survival (OS) across age groups [29,30].

### 2.2. Intrinsic Tumor Resistance

#### 2.2.1. Antigen Escape

Following CAR T-cell therapy, various mechanisms can lead to resistance through antigen escape, characterized by reduced expression or complete loss of the target antigen on the cell surface. These mechanisms include genetic alterations, epigenetic modifications, and clonal selection, as well as antigen shedding and internalization. This phenomenon is influenced by several factors, including prior antigen-targeted immunotherapies and the biology of the underlying disease [31,32].

A common cause of antigen escape is the alteration to the DNA sequence encoding the antigen. For example, there is evidence that loss of CD19 or BCMA is frequently associated with homozygous biallelic frameshift mutations. When only a monoallelic mutation is detected, additional mechanisms, such as silencing of the second allele, are likely contributing to antigen loss [33,34]. Another mechanism of CD19 loss involves aberrant CD19 processing through alternative splicing. Hundreds of CD19 isoforms can arise from single-point mutations or cryptic splice sites, leading to resistance to CAR T-cell therapy [35,36]. Similar alterations can affect various exons of the CD19 gene, resulting in protein misfolding (e.g., exon 2) or disrupted membrane anchoring (e.g., exons 5 and 6). In some cases, antigen expression is altered through intron retention; for instance, introns 2 and 6 are more abundantly retained in patients who develop CAR T-cell resistance, even prior to treatment [37,38]. Genetic alterations such as RNA fusions, cryptic splicing, frameshifts, or biallelic deletions can also affect the *MS4A1* gene encoding the protein CD20, similarly leading to antigen loss [39]. These diverse genetic changes ultimately result in complete antigen loss on the cell membrane or in the production of antigen isoforms that fail to bind to the CAR [40,41]. Finally, CD19 expression can also be downregulated through epigenetic modifications, such as promoter hypermethylation [42,43]. In addition, antigen loss may also result from the disruption of other proteins involved in antigen expression such as CD81 deletion, observed in B-ALL patients who relapse with CD19-negative disease [44]. Ziccheddu et al. [45] investigated 54 LBCL patients treated with CAR T-cells using whole-genome and RNA sequencing. Their findings demonstrated that CD19 downregulation can be associated with loss-of-function mutations in *PAX5*, *EBF1*, and *RHOA*, key regulators of CD19 expression.

At any point in their history, from diagnosis to subsequent lines of therapy, cancers may exhibit varying degrees of clonal heterogeneity, including a subset of antigen-negative clones. For instance, approximately 20% of B-ALL cases harbor CD19 and CD22 negative subclones which, under the selective pressure of CAR T-cell therapy, can expand and cause treatment resistance [46].

Antigen escape can also occur following treatment with anti-BCMA CAR T-cells, although BCMA-negative clonal relapses appear less frequent, occurring in fewer than 5% of cases compared to the 9–24% reported in B-ALL and 20–27% in B-NHL patients [4,5,19,47,48,49]. This discrepancy is likely due to the essential role of BCMA in plasma cell survival [50,51]. Mechanisms driving BCMA loss include biallelic deletion of the BCMA encoding gene *TNFRSF17*, monoallelic loss combined with additional alterations to the second allele, or in-frame deletions affecting the extracellular domain of BCMA [41]. Notably, CAR T-cell-resistant MM often exhibits substantial intratumoral heterogeneity, with the frequent coexistence of both antigen-positive and antigen-negative clones [52]. Similarly, a study of a small cohort of MM patients relapsing after a *GPRC5D*-targeted CAR T-cell therapy revealed diverse mechanisms of antigen loss or reduction: one-third of patients exhibited genetic deletions, while the majority showed hypermethylation affecting the transcriptional regulation of *GPRC5D* [53].

Epitope masking, which can occur through the unintended transduction of leukemic B-cells with the CAR construct during the manufacturing process, is another reported mechanism of antigen escape. In this scenario, the CAR molecule is expressed on the surface of the malignant cell and binds its target antigen in cis, thereby masking it and protecting the cell from recognition. This phenomenon, although rare, can be avoided with strict manufacturing procedures [54].

#### 2.2.2. Trogocytosis

Trogocytosis is a cell-contact-dependent, bidirectional transfer of membrane and cytoplasmic material between cells. It was first identified in studies of the immunological synapse between T-cells and antigen-presenting cells, where it emerged as a key mechanism of immune activation and adaptability [55]. This process involves various immune cell lineages and has been extensively studied in T- and natural killer (NK)-cells, revealing both stimulatory and inhibitory effects on immune responses depending on the context [56]. Trogocytosis also affects CAR T-cells and can contribute to treatment resistance by mediating the transfer of target antigens from tumor cells to CAR T-cells. This leads to reduced antigen density on malignant cells and to fratricidal killing of CAR T-cells that have acquired the tumor antigen [57,58]. Moreover, trogocytosis may promote functional exhaustion of CAR T-cells, as evidenced by increased expression of inhibitory receptors such as PD-1 and LAG-3 [59]. The extent of this resistance mechanism is influenced by multiple factors, including antigen density, CAR binding affinity, checkpoint receptor expression, and downstream intracellular signaling [60]. While the regulatory mechanisms of trogocytosis remain incompletely understood, emerging evidence points to tumor-derived factors as key drivers of this process, particularly through the modulation of cholesterol 25-hydroxylase and transcriptional regulators such as ATF3 [61]. Other important modulators of trogocytosis include antigen-binding affinity and the composition of CAR costimulatory domains. Lower-affinity CARs have been shown to reduce trogocytosis, offering a potential strategy to mitigate antigen loss. Additionally, certain costimulatory domains, such as 4-1BB, appear to enhance trogocytosis [62].

#### 2.2.3. Lineage Switch

Targeted therapies exert strong immunological selective pressure on tumor cells, triggering complex adaptive mechanisms including lineage switch. This phenomenon consists in the emergence of resistant neoplastic clones with a distinct phenotype, due to a profound transformation of the malignant clone, driven by extensive cellular reprogramming through alterations in key transcription factors. Lineage switch often results in the loss of the target antigen [63]. It has been extensively described in B-ALL with *KMT2A* rearrangements and is driven by the inherent genetic instability of these diseases. The switch often results in a transition to a myeloid or mixed lymphoid–myeloid phenotype [64,65,66]. Although less frequent, lineage switching has also been reported in B-ALL patients with *CRLF2* rearrangements or Philadelphia chromosome positivity [67,68]. Additional rare cases of lineage switching include CLL transforming into plasmablastic lymphoma and MCL undergoing transdifferentiation into sarcoma following anti-CD19 CAR T-cell therapy [69,70].

#### 2.2.4. Tumor Genetic Alterations

Among the genomic alterations associated with poor therapeutic outcomes, several have been specifically linked to resistance or suboptimal response to CAR T-cell therapy. Genes involved in apoptosis regulation, such as *FAS* (reduced expression) and *BCL2* (upregulation), play a central role and have been correlated with poor clinical responses [71,72,73]. As expected, *TP53* mutations are also associated with poor responses, potentially impairing CAR T-cell efficacy through dysregulation of apoptosis pathways, interferon signaling, and impaired immune cell infiltration [74]. As demonstrated by Cox et al. [75], *TP53* mutations may contribute not only to antigen loss but also to antigen-independent resistance by downregulating death receptors such as Fas (CD95) and DR5 (TNFRSF10B) [73,76].

Somatic mutations in class I human leukocyte antigen (HLA) genes are frequently observed in LBCL patients who relapse after CAR T-cells. However, only those with biallelic loss of the HLA class I complex exhibit significantly reduced PFS. While such alterations would typically be expected to trigger NK-cell-mediated tumor clearance, concurrent mutations in genes regulating NK-cell cytotoxicity, such as *D58*, *CD48*, *MICA*, *PVR*, *SPPL3*, and *TNFSF9* (loss of function), and *MUC1*, *GMDS*, and *CD44* (gain of function), appear to impair this compensatory mechanism [45]. These findings underline that CAR T-cell resistance is intricately linked not only to escape from adaptive immunity but also from innate immune responses.

#### 2.2.5. Tumor Microenvironment

The efficacy of CAR T-cell therapy is further compromised by a profoundly altered tumor microenvironment (TME) which supports neoplastic growth and hinders antitumor immunity through multiple mechanisms.

The hypoxic nature of the neoplastic microenvironment induces the constitutive production of pro-angiogenic factors (VEGF, PDGF, FGF, TGF-β), resulting in rapid formation of aberrant vascular networks. Endothelial cells exhibit reduced expression of adhesion molecules (VCAM1, ICAM1), thus impeding cellular extravasation, including extravasation and trafficking of CAR T-cells. The pro-inflammatory TME promotes the recruitment of cancer-associated fibroblasts, which typically express high levels of fibroblast activation protein and continuously deposit and remodel the extracellular matrix, further hampering T-cell infiltration [77,78]. TGF-β plays a particularly important role: secreted by a multitude of stromal and immune cells, it inhibits CD8^+^ T-cell functionality through transcriptional downregulation of genes encoding granzymes, perforins, and cytotoxins. It also promotes differentiation of CD4^+^ T-cells into regulatory T-cells (T_regs_) and has been implicated in the expansion, survival, and immune evasion of MM cells within the bone marrow niche [79,80,81]

Moreover, the TME can be enriched in CD4^+^ T_regs_, which secrete additional immunosuppressive cytokines such as IL-10 and TGF-β, as well as myeloid-derived suppressor cells (MDSCs) and M2-polarized TAMs (tumor-associated macrophages), all of which are skewed towards an immunosuppressive phenotype known to promote tumor proliferation. Additionally, increased macrophage infiltration at the tumor site has been associated with less durable responses to some CAR T-cell products [82,83,84,85,86]

Tumor hypoxia and necrosis further increase concentrations of immunosuppressive metabolites such as adenosine and lactate, leading to metabolic acidosis, reduced IFN-γ production, impaired T-cell function, M2 polarization of TAMs, and upregulation of PD-L1 expression [87,88,89,90]

The oncometabolite kynurenine, a product of tryptophan catabolism by IDO1 (Indoleamine-2,3-Dioxygenase 1) or TDO2 (Tryptophan-2,3-Dioxygenase 2), is present at high concentrations in the TME and directly inhibits glucose uptake by T-cells, leading to diminished cytotoxicity capacity and proliferation. Tryptophan depletion also deprives CAR T-cells of an essential amino acid. Interestingly, fludarabine and cyclophosphamide—commonly used in lymphodepleting regimens—have been shown to inhibit intracellular IDO expression, thus hypothetically enhancing the activity of CAR T-cells [91]

A TME rich in IFN-γ and infiltrated with MDSCs plays a key role in hampering CAR T-cell expansion, as observed in patients with LBCL treated with axi-cel. Chronic IFN-γ stimulation leads to upregulation of inhibitory ligands (PD-L1, PD-L2, Galectin-9, HHLA2, VISTA) via increased transcription of multiple genes commonly associated with T-cell exhaustion and inferior CAR T-cell response quality. This highly pro-inflammatory TME may be indirectly evaluated by pre-infusion serum levels of ferritin, IL-6, and CRP [92]

Finally, the PD1/PD-L1 axis, overexpressed within the TME and partly driven by IFN-α, IFN-β, and IFN-γ, plays a central role in inducing T-cell anergy through physiological mechanisms of self-tolerance and immune modulation. PD1 and CTLA4 upregulation occurs upon antigen encounter, while tumor cells are known to upregulate PD-L1 in response to T-cell cytokine release. Interaction between these actors—normally expressed on T-, B-, NK-cells, and macrophages—ultimately inhibits both innate and adaptive immunity in PD1+ cells through signaling pathways involving the TCR or BCR [93,94,95]

#### 2.2.6. Other Issues: CAR-Positive Relapses

Another critical concern at the time of leukapheresis is the inadvertent collection and subsequent transduction of malignant B-cells, which may result in so-called CAR-positive relapses. This phenomenon, first described by Ruella and colleagues [54], highlights the risk that a leukemic clone could integrate the CAR transgene and thereby evade therapy. To reduce this risk, specific enrichment steps for T-lymphocytes have been incorporated into certain manufacturing platforms, such as brexu-cel, which includes additional T-cell selection procedures to minimize malignant B-cell contamination and ensure the integrity of the final product [8].

### 2.3. Tumor-Independent Mechanisms of Resistance: Role of the Microbiota

Antibiotic-induced microbiome dysbiosis—the disruption of the non-pathogenic gut microbiota caused by antibiotic use—has emerged as a significant factor associated with poorer outcomes in several antineoplastic settings, including immune checkpoint inhibitors, allogeneic stem cell transplantation, and CAR T-cell therapy [96,97,98,99,100,101]. In a study involving over two hundred patients with B-NHLs or B-ALL, exposure to antibiotics—particularly piperacillin/tazobactam and carbapenems—within 4 weeks prior to CAR T-cell infusion was associated with reduced gut microbiota diversity, decreased OS, and higher incidence of toxicities such as immune-effector-cell-associated neurotoxicity syndrome (ICANS) and cytokine release syndrome (CRS). Conversely, a higher abundance of obligate anaerobic bacteria in stool samples, such as *Ruminococcus*, *Bacteroides*, and *Faecalibacterium*, was linked to improved CR rates and a lower incidence of toxicity [99]. These findings were further supported and expanded by two large-scale studies. In a cohort of 172 patients with B-NHLs, exposure to “high-risk” antibiotics—including piperacillin/tazobactam, cefepime, ceftazidime, and meropenem—within 3 weeks prior to CAR T-cell infusion was significantly associated with shorter PFS and increased incidence of ICANS of any grade, although no association with CRS was observed. Moreover, specific associations were identified between the composition of gut microbiota, CAR T-cell product characteristics, and therapy outcomes: *Bifidobacterium longum* and peptidoglycan biosynthesis—a bacterial pathway often upregulated in antibiotic-resistant strains—strongly correlated with long-term survival and CAR T-cell response, independently of clinical or demographic variables. *Lachnospira pectinoschiza* and *Akkermansia muciniphila* were significantly associated with CD3^+^ and CD4^+^ T-cell counts at the time of apheresis, whereas *Bacteroides*, *Blautia*, and *Faecalibacterium prausnitzii* were negatively correlated with CD3^+^ and CD8^+^ T-cell levels [101]. Similarly, in a study of 422 patients with LBCL, exposure to antibiotics targeting commensal anaerobic gut bacteria—specifically piperacillin/tazobactam and carbapenems—before CAR T-cell infusion was associated with a worse outcome. Notably, a decrease in short-chain fatty acids (SCFAs) and other anionic metabolites was observed, proportional to the reduction in the abundance of multiple bacterial species in fecal samples—including *Roseburia*, *Faecalibacterium prausnitzii*, *Ruminococcus* spp., *Bifidobacterium*, *Bacteroides*, and *Akkermansia muciniphila*. Importantly, SCFAs were shown to enhance the metabolic fitness of CAR T-cells and improve their tumor-killing capacity [100]. On this basis, interventional approaches are now under clinical investigation, with ongoing trials evaluating the use of fecal microbiota transplantation (FMT) to restore microbial diversity and improve outcomes in patients receiving CAR T-cell therapy (NCT06218602, NCT07042438).

**Figure 1:**
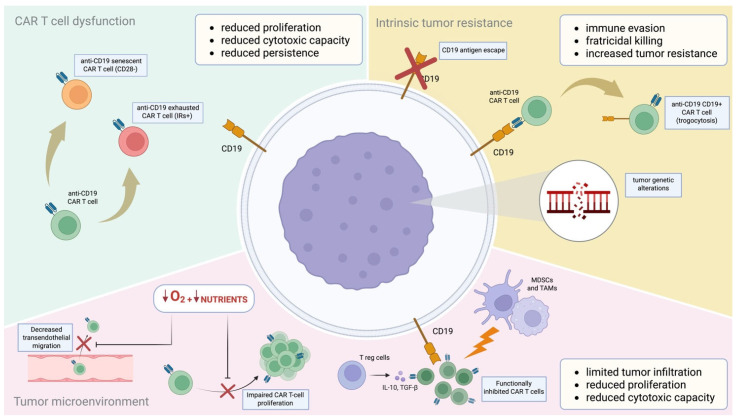
Key mechanisms of resistance to CAR T-cell therapy. Resistance to CAR T-cell therapy arises from multiple, often overlapping, mechanisms. These include CAR T-cell dysfunction, characterized by progressive loss of proliferative capacity, cytotoxic function, and persistence due to senescence or exhaustion (top left); intrinsic tumor resistance, involving antigen escape (e.g., loss or downregulation of CD19), trogocytosis-mediated fratricidal killing, and tumor-intrinsic genetic alterations enhancing immune evasion (top right); and an immunosuppressive tumor microenvironment, which impairs CAR T-cell trafficking, expansion, and activity through hypoxia, nutrient deprivation, suppressive cytokines (e.g., IL-10, TGF-β), regulatory T-cells (Tregs), myeloid-derived suppressor cells (MDSCs), and tumor-associated macrophages (TAMs) (bottom). Together, these factors contribute to limited tumor infiltration, reduced cytotoxic capacity, and diminished therapeutic efficacy.

The main mechanisms of resistance to CAR T-cell therapy are summarized in Figure 1.

## 3. Overcoming CAR T-Cell Resistance

### 3.1. CAR Engineering

#### 3.1.1. CAR Modifications

**Figure 2:**
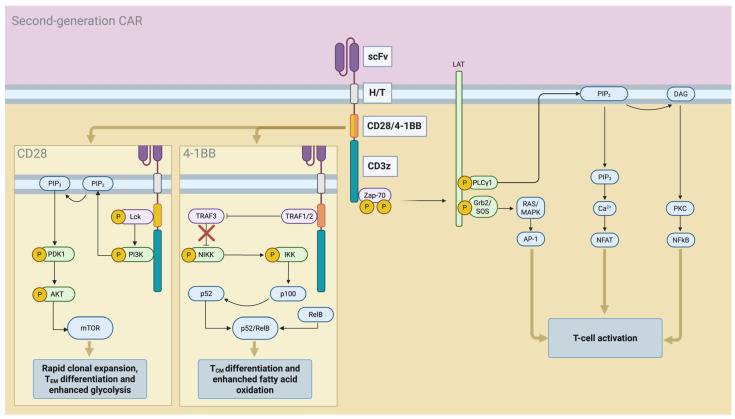
Structure and signaling pathways of second-generation CAR T-cells. Upon antigen recognition, CAR engagement triggers CD3z phosphorylation and recruitment of ZAP-70, leading to downstream LAT signaling and activation of NFAT, NF-κB, and AP-1 transcription factors. Distinct costimulatory domains promote divergent signaling and metabolic programs. CD28 costimulation recruits Lck and PI3K, driving PDK1/AKT/mTOR activation, robust clonal expansion, T_EM_ differentiation, and glycolytic reprogramming. In contrast, 4-1BB costimulation recruits TRAF1/2, leading to TRAF3 degradation, NIK stabilization, and activation of the non-canonical NF-κB pathway (p52/RelB), which supports T_CM_ differentiation, enhanced fatty acid oxidation, and long-term persistence. Abbreviations: H/T (hinge/transmembrane), scFv (single-chain variable fragment), T_CM_ (central memory T-cells), T_EM_ (effector memory T-cells).

First-generation CAR T-cells were designed with a CAR comprising a single-chain variable fragment (scFv), responsible for antigen recognition, a hinge/transmembrane (H/T) domain, and an intracellular CD3z domain containing three immunoreceptor tyrosine-based activation motifs (ITAMs), crucial for initiating T-cell activation. However, these constructs demonstrated limited efficacy, mainly due to poor proliferative capacity and insufficient persistence. To overcome these limitations, second-generation CAR T-cells were engineered to include a costimulatory domain—typically CD28 or 4-1BB—inserted between the transmembrane region and the CD3z domain. The addition of this “second signal” enhanced both the potency and durability of responses, leading to the FDA approval of the first second-generation CAR T-cell product, tisa-cel, in 2017 for R/R B-ALL [102]. The structure and signaling pathway of second-generation CAR T-cells is summarized in Figure 2.

Considerable efforts have been made to further refine CAR design in order to enhance efficacy and overcome mechanisms of resistance. One of the earliest areas of interest was comparing the performance of the two most commonly used costimulatory domains, CD28 and 4-1BB. Data from pivotal clinical trials, including ELIANA and ZUMA-1, highlighted significant functional differences: CAR T-cells with 4-1BB costimulation showed slower expansion kinetics but longer persistence, while CD28-containing CARs expanded more rapidly but persisted for a shorter duration [1,4]. Although the precise molecular basis underpinning these differences is not yet fully elucidated, mechanistic studies have shed light on distinct signaling and metabolic profiles. CD28-based CARs (28z CARs) tend to produce higher levels of IL-2 and IFN-γ, favoring the acquisition of a T_EM_ phenotype and a more glycolytic metabolic state. In contrast, 4-1BB-based CARs (BBz CARs) are characterized by more moderate cytokine production, differentiation into T_CM_, and enhanced fatty acid oxidation [103,104]. These differences can be partially attributed to the ability of 4-1BB to activate non-canonical NF-κB signaling, recruit TNF-receptor-associated factors (TRAFs), promote Th1 polarization and IL-21 secretion, and downregulate PD-1 expression [105,106,107]. In contrast, CD28’s constitutive association with the tyrosine kinase Lck leads to higher basal phosphorylation, stronger and more immediate signaling, increased IL-2 production—which can inadvertently promote T_reg_ infiltration in the tumor microenvironment—and reduced CAR T-cell persistence [108,109]. Remarkably, deletion of the Lck-binding motif within the CD28 endodomain eliminated IL-2 secretion, reduced T_reg_ recruitment, and enhanced persistence without compromising cytotoxicity or IFN-γ release [109].

These findings suggest that the robust activation conferred by CD28-based CARs may paradoxically lead to early exhaustion and impaired persistence. To address this, CARs with attenuated signaling strength—termed “tuned CARs”—have been developed by modulating the number of ITAMs in the CD3z domain. Notable examples include CD28z 1XX (bearing a single proximal ITAM) and CD28z XX3 (bearing a single distal ITAM). While both retained in vitro cytotoxicity comparable to conventional 28z CARs, CD28z 1XX CARs demonstrated superior in vivo persistence and T_CM_ differentiation, translating into prolonged survival. Conversely, CD28z XX3 CARs were linked to rapid disease progression, underscoring the importance of ITAM positioning [110].

Aligned with the “less activation = more persistence” paradigm, low-affinity CARs have also been developed. These constructs exhibit approximately 40-fold reduced binding affinity to their target antigen and offer two key advantages beyond prolonged persistence: diminished trogocytosis and reduced fratricide of CAR T-cells that acquire the antigen through trogocytosis [62]. In the phase I CARPALL trial, low-affinity anti-CD19 CARs (CAT CARs) achieved high response rates and prolonged survival in pediatric patients with R/R B-ALL [111]. Building on this concept, obecabtagene autoleucel (obe-cel), an autologous 4-1BB-based anti-CD19 CAR T-cell therapy incorporating a fast off-rate scFv with intermediate affinity, was evaluated in the phase Ib/II FELIX trial in adults with R/R B-ALL. Obe-cel demonstrated a 77% ORR, a median event-free survival (EFS) of 11.9 months, and low rates of grade ≥3 CRS (2,4%) and ICANS (7.1%) [112]. However, both tuned and low-affinity CARs may be less effective against tumor cells with low antigen density—a potential mechanism of immune escape, especially for BBz CARs. To overcome this limitation, BBzz CARs—CARs containing a 4-1BB costimulatory domain and two CD3z chains—were designed, achieving efficacy against low-antigen-density targets comparable to 28z CARs while retaining the superior persistence of BBz CARs [113].

Modifications of the H/T domain have also been explored. The improved performance of 28z CARs against low-antigen targets has been partly attributed to the CD28-derived H/T region, which facilitates a more stable and organized immunological synapse than the CD8-derived H/T found in BBz CARs. Thus, substituting the CD28 H/T into BBz CARs improved their activity against low-antigen-density cells to levels similar to 28z CARs [113]. More recently, alternative H/T domains such as CD1a have been tested. In a comparative study, CD1a-derived H/T domains led to reduced surface CAR expression (due to enhanced internalization and recycling), dampened cytokine production, lower exhaustion, enhanced T_CM_ differentiation, and improved tumor control [114].

Another strategy involved engineering novel scFvs targeting alternative CD19 epitopes. AT101 CARs, based on the h1218 scFv—which binds a more membrane-proximal epitope than the canonical FMC63—were effective against FMC63-resistant models, including those with epitope masking or point mutations. AT101 CARs exhibited superior on/off kinetics, enhanced expansion, reduced exhaustion, and better tumor control, as revealed by early studies on R/R B-NHLs [115].

Finally, advances in non-viral gene delivery platforms and genome editing technologies have significantly expanded the repertoire for CAR T-cell engineering. DNA transposon systems, such as Sleeping Beauty and piggyBac, have emerged as cost-effective, virus-free alternatives for stably integrating CAR constructs into T-cells. These platforms allow for the delivery of large transgene cassettes with sustained CAR expression and have shown promising results in preclinical and early-phase clinical studies [116,117]. However, concerns have been raised regarding potential insertional mutagenesis, especially with piggyBac, which has been associated with integration near oncogenes and rare cases of lymphomagenesis [118,119]. In parallel, genome editing strategies using the CRISPR/Cas9 system have enabled the precise insertion of CAR transgenes into specific genomic loci. A landmark study by Eyquem et al. [120] demonstrated that targeting the CAR construct to exon 1 of the TRAC gene—which encodes the T-cell receptor alpha constant region—resulted in uniform CAR expression, reduced tonic signaling, enhanced receptor recycling, improved T_CM_ differentiation, reduced exhaustion, and superior tumor control in murine models.

#### 3.1.2. Alternative Costimulatory Domains and Third-Generation CAR T-Cells

Beyond the canonical CD28 and 4-1BB costimulatory domains, several alternative domains have been investigated with the aim of optimizing the effector function and persistence of CAR T-cells. Among these, costimulatory domains such as CD27, OX40, ICOS, and IL-15Rα have demonstrated promising results [121,122,123,124].

In parallel with efforts to explore alternative costimulatory domains, third-generation CAR T-cells have been developed. These constructs incorporate two costimulatory domains (e.g., CD28/OX40, ICOS/4-1BB) within the same CAR molecule, with the goal of combining the functional benefits of each. For example, co-expression of CD28 and OX40 was designed to pair the robust initial activation provided by CD28 with OX40’s ability to sustain T-cell proliferation and survival. In vitro models of neuroblastoma showed that these third-generation CARs retained cytotoxic activity comparable to that of first- and second-generation CARs while displaying enhanced expansion and persistence, even under repeated antigen stimulation [125]. Another example is the ICOS/4-1BB third-generation CAR, evaluated in preclinical models of solid tumors. These constructs exhibited superior antitumor activity compared to second-generation CARs, with reduced tonic signaling and improved persistence. [126].

#### 3.1.3. Dual Targeting

One promising strategy to address disease relapse driven by CD19 antigen escape is dual antigen targeting, which involves incorporating an additional CAR that is specific for an alternative antigen (e.g., CD22 or CD20). Several approaches have been developed to implement this strategy, including co-administration of monospecific CAR T-cells, cotransduction of two CAR constructs, bicistronic vectors, and tandem or loop CAR architectures.

Co-administration of monospecific CAR T-cells targeting CD19 and CD22 has shown high response rates in B-cell malignancies. In a phase I trial on heavily pretreated patients with B-ALL, the combined infusion of two distinct CAR T-cell products resulted in 100% CR with minimal residual disease (MRD) negativity in all evaluable patients, with most responses proving durable [127]. These findings were confirmed in a larger phase II trial involving 225 pediatric patients [128]. Similarly, a pilot study of 89 patients with CD19^+^CD22^+^ R/R malignancies also reported high efficacy [129]. Notably, relapses were rarely associated with CD22 antigen escape. This observation can be explained by the lower selective pressure exerted by anti-CD22 CARs, as CD22 is expressed at lower and more heterogeneous levels compared to CD19, undergoes rapid internalization upon receptor engagement, and exerts weaker signaling thresholds, resulting in reduced immunologic pressure [130,131,132]. Consequently, in dual CD19/CD22 CAR approaches, immune evasion predominantly occurs through CD19 loss rather than CD22 negativity.

In MM, co-administration of anti-BCMA and anti-CD19 CAR T-cells demonstrated encouraging results. In a phase II trial, this approach yielded an ORR of 92% and a CR rate of 60%, with a median PFS of 18.3 months. These promising outcomes may reflect the elimination of CD19^+^ “myeloma stem-like” cells and a reduced risk of BCMA-negative relapse [133,134].

Co-transduction involves transducing T-cells with two separate viral vectors encoding different CARs, resulting in a heterogeneous population in which only a fraction co-express both receptors. In preclinical B-ALL models, anti-CD19/CD22 co-transduced CAR T-cells effectively eliminated both single- and dual-antigen-positive tumor cells in vitro and cleared CD19^−^ tumors in vivo [135]. This strategy was evaluated clinically in the phase I CARPALL trial in 12 pediatric patients with R/R B-ALL, achieving an 83% MRD-negative CR rate with no cases of antigen escape [136].

Bicistronic CAR constructs, which use a single vector to encode two CARs (each with a distinct scFv), offer an alternative approach. AUTO3, a bicistronic anti-CD19/CD22 CAR, was evaluated in the phase I AMELIA trial, inducing an 80% MRD-negativity in 15 pediatric and young adult patients with R/R B-ALL. However, relapse occurred in nine patients, eight of whom had low CAR T-cell levels, suggesting poor persistence as the primary mechanism of relapse—potentially inferior to that observed with second-generation CAR T-cells [137].

Tricistronic constructs targeting CD19, CD20, and CD22, were found to be effective in vitro and in vivo against CD19^−^ primary B-ALL models and patient-derived ex vivo samples of patients relapsed after anti-CD19 CAR T-cells [138].

Despite their efficacy, co-transduction and bicistronic CARs present certain limitations. Qin et al. [139] reported that only ~25% of co-transduced products co-expressed both CARs, with preferential expansion of CD19-only CAR T-cells, undermining dual targeting efficacy. Similarly, Cordoba et al. [137] observed imbalanced CAR expression in bicistronic products, with anti-CD19 CARs typically dominating. To address these issues, tandem (TanCARs) and loop (LoopCARs) CARs were developed, wherein two scFvs are combined in a single polypeptide chain. TanCARs arrange the two scFvs linearly, while LoopCARs interleave the VH and VL regions of each scFv, enhancing folding, surface expression, and dual antigen recognition [139].

A CD19/CD20-targeting TanCAR (LV20.19 or zamtocabtagene autoleucel) tested in a phase I trial for R/R B-NHL achieved a 100% ORR and 92% CR rate in patients treated at the target dose, and preliminary data of phase II studies confirmed deep and durable responses in R/R LBCL setting [140,141]. Another CD19/CD20 TanCAR (TanCAR7) evaluated in a phase I/II trial on R/R B-NHL patients yielded a 70% CR, with a median PFS of 27.6 months and median OS not reached. Notably, most patients previously treated with anti-CD19 CAR T-cells responded, and only 1 out of 12 relapsed patients who underwent biopsy exhibited dual antigen loss (CD19^−^/CD20^−^), indicating resistance to antigen escape [142]. In MM, a TanCAR targeting BCMA and transmembrane activator and CAML interactor (TACI) showed activity against BCMA-negative models in vitro [143].

LoopCARs targeting CD19 and CD22 demonstrated efficacy against CD19^+^CD22^+^, CD19^−^CD22^+^, and CD19^+^CD22^−^ tumors in preclinical models [139]. Preliminary studies showed high rates of deep response in patients with R/R B-ALL and LBCL but emphasized the need to optimize persistence and expansion, both inferior to those achieved with monospecific anti-CD22 CARs. Consistent with other CD19/CD22 bispecific CARs, relapses were linked to CD19 loss rather than CD22 escape [144,145].

Beyond CD19, CD22, and CD20, a number of alternative antigens have been explored in preclinical dual-CAR platforms. Dual-specific CARs targeting CD19/CD79b, CD19/CD38, CD19/CD123, and CD19/CD37 have shown promising anti-tumor activity in vitro and in vivo [146,147,148,149,150,151].

#### 3.1.4. Fourth-Generation CAR T-Cells: Armored CARs and CAR TRUCKs

Fourth-generation CAR T-cells represent a significant evolution beyond conventional CAR T-cell platforms. These advanced constructs are engineered not only to express a CAR but also to produce additional bioactive molecules—such as cytokines, checkpoint-blocking minibodies, or switch receptors—that enhance antitumor activity, counteract the immunosuppressive TME, and prolong T-cell persistence. These enhanced CAR T-cells are commonly referred to as armored CAR T-cells or T-cells redirected for universal cytokine killing (CAR TRUCKs) [102].

CAR TRUCKs represent a specialized subset of fourth-generation CAR T-cells engineered to secrete cytokines either constitutively or in an antigen-inducible manner, which exert both autocrine and paracrine effects: they promote T-cell cytotoxicity, persistence, and central memory differentiation while simultaneously activating components of the host immune system, including NK cells, dendritic cells, and endogenous non-transduced T-lymphocytes. In preclinical models, certain CAR TRUCKs have demonstrated therapeutic efficacy even in the absence of lymphodepleting chemotherapy, offering a potential strategy for patients ineligible for standard conditioning regimens [152,153,154,155].

Armored CAR T-cells have been developed to overcome immunosuppressive signals within the TME and improve in vivo persistence. A widely explored strategy involves engineering CAR T-cells to secrete checkpoint-blocking minibodies, such as those targeting PD-1, CTLA-4, or TIM-3. These molecules mediate local immune checkpoint blockade and have shown comparable or superior efficacy with reduced systemic toxicity compared to systemic checkpoint inhibitors like pembrolizumab [156,157]. Another promising approach involves the use of switch receptors, chimeric proteins that convert inhibitory signals into activating ones by replacing the intracellular inhibitory domain (e.g., from PD-1 or FAS) with a costimulatory domain such as CD28 or 4-1BB. This strategy has yielded encouraging results in preclinical models and early clinical trials in R/R B-NHLs [158,159,160]. Similarly, dominant negative receptors have been designed to act as decoys: they bind immunosuppressive ligands (e.g., PD-L1 or FASL) but lack intracellular signaling domains, thereby neutralizing inhibitory cues without transmitting suppressive signals [161,162].

Advances in gene editing have further expanded the toolkit for enhancing CAR T-cell function. For instance, forced expression of the transcription factor JUN in CAR T-cells (JUN-HA-28z) counteracts IRF4/BATF-driven exhaustion, leading to enhanced antitumor efficacy, proliferation, and long-term persistence [163]. CRISPR-based genetic screens have also identified targetable genes whose disruption enhances CAR T-cell function, such as *RASA2*, *SOCS1*, *TCEB2* (ELOB), *CBLB*, and *SUV39H1,* leading to improved cytotoxicity, memory differentiation, and in vivo persistence [164,165,166,167]. Complementary to genome editing, post-transcriptional checkpoint silencing has been explored through a dual-short/small harpin RNA (shRNA) approach targeting PD-1 and TIGIT. This method employs a “two-in-one” lentiviral vector integrating both the CAR construct and two shRNA cassettes for simultaneous checkpoint inhibition and is currently under clinical investigation in a phase 1–2 trial for R/R LBCL [168]. Epigenetic modulation has also emerged as a viable strategy for CAR T-cell enhancement. Inhibition of key regulators such as LSD1 and Nrf2 has been shown to influence T-cell differentiation, memory programming, and effector function, further contributing to durable responses [169,170].

Additional approaches have focused on cell survival and proliferation. For instance, bicistronic vectors have been used to co-express anti-apoptotic proteins like BCL-2 or BCL-XL or constitutively active IL-7 receptors, thereby improving the persistence and expansion of CAR T-cells [171,172].

Other innovations have targeted intracellular trafficking and receptor recycling. Overexpression of Rab5 GTPase, a key regulator of early endosome formation, can prevent a process termed “CAR-jacking,” in which tumor cells internalize and degrade CAR molecules [173]. Similarly, CAR constructs lacking lysine residues in the cytoplasmic domain (CARKRs) are resistant to ubiquitination and lysosomal degradation, promoting receptor recycling and sustained surface expression [174].

A particularly novel and highly specific platform is the SEAKER system: CAR T-cells are engineered to secrete enzymes that activate systemically administered prodrugs within the TME, enabling antigen-independent cytotoxicity while limiting systemic toxicity [175].

Finally, the CAR BiTE platform allows CAR T-cells to secrete bispecific T-cell engagers (BiTEs), thereby recruiting and activating bystander T-cells. This strategy has shown efficacy in preclinical models of solid tumors with heterogeneous antigen expression, including glioblastoma [176].

#### 3.1.5. Boolean and Conditional Logic in CAR T-Cells

Boolean logic has been increasingly applied to CAR T-cell engineering to enhance specificity and safety. In “AND-gate” designs, full activation requires recognition of two antigens, thereby restricting cytotoxicity to dual-positive tumor cells. “OR-gate” constructs, such as tandem, loop, or bicistronic CARs, trigger activation upon recognition of either target, reducing the risk of antigen escape. “NOT-gate” circuits employ inhibitory CARs that suppress activation when encountering antigens expressed on healthy tissues, thus mitigating on-target/off-tumor toxicity. Beyond these classical Boolean rules, conditional IF/THEN circuits based on SynNotch receptors enable sequential decision-making: engagement with a priming antigen (“IF”) induces expression of a secondary CAR or effector program (“THEN”), thereby confining activity to cells with the correct antigenic context. More recently, an “IF-BETTER” logic has been proposed, in which CAR recognition of a primary antigen is potentiated—but not strictly dependent—on a secondary input delivered through a costimulatory chimeric receptor. Overall, both Boolean-logic-based and conditional signal CAR designs have demonstrated promising activity in preclinical models, showing enhanced specificity, cytokine production, persistence, and tumor control across different tumor settings [177]. Notably, despite encouraging preclinical results, no clinical trials evaluating Boolean- or conditional-logic-based CAR T-cell designs are currently ongoing.

### 3.2. T-Cell Collection, Selection, and Manufacturing

Significant differences in CAR T-cell performance and manufacturing outcomes appear to depend not only on the underlying disease but also on the timing and method of collection, as well as on the characteristics of the transduced T-cell population.

As discussed before, commercially available CAR T-cell products are typically derived from unfractioned (T_BULK_) T-cell populations [22]. Failed manufacturing batches often contain more mature and differentiated T-cells, whereas higher success rates are associated with larger quantities of less mature or differentiated T-cells expressing elevated levels of CD25. Selecting differentiated T-cells negatively impacts antitumor efficacy, while early memory and naïve T-cells are considered optimal due to their enhanced persistence and reduced propensity for exhaustion. Among these, T_SCM_ are particularly promising thanks to their plasticity and self-renewal capacity. Although rare in peripheral blood, T_SCM_ can be enriched in vitro by stimulating naïve T-cells with IL-7 and IL-15. In preclinical models, a CAR T-cell product generated from pre-selected naïve/stem memory T-cells (CAR T_N/SCM_) demonstrated superior antitumor activity, a more favorable toxicity profile, and a reduced acquisition of exhausted-like phenotypes compared to the CAR T_BULK_ product [23,178,179]. Joedicke et al. [180] demonstrated that selecting specific T-cell populations, though technically challenging and resulting in smaller yields, allows for the successful generation of anti-BCMA CAR T-cells enriched in T_SCM_ and central memory phenotypes. Enhanced activation and effector function have been linked to higher risks of toxicities such CRS and ICANS; however, T_SCM_ and naïve T-cells support increased expansion with lower intracellular activation signaling, potentially offering a more balanced activity with strong antitumor effects and reduced toxicity [23].

Other studies have shown that prior chemoimmunotherapy can negatively affect T-cell quality, with patients collected after ≥2 treatment lines displaying greater exhaustion and reduced proliferative capacity despite similar lymphocyte counts [181,182]. Adequate chemotherapy washout before leukapheresis is therefore critical. In particular, recent bendamustine exposure has been linked to impaired T-cell collection, lower proliferative potential, and reduced manufacturing success, leading current recommendations to advise its avoidance—together with other lymphotoxic agents such as fludarabine, cladribine, and pentostatin—whenever feasible [183,184,185]. Corticosteroid washout is also required, as their immunosuppressive and pro-apoptotic effects can transiently impair collection efficiency. Expert consensus suggests discontinuing systemic steroids at least 72 h, and ideally 7 days, before leukapheresis, with recovery of absolute lymphocyte count ≥0.2 × 10^9^/L [185]. By contrast, T-cell-sparing bridging strategies—such as monoclonal antibodies, brief corticosteroid courses with adequate washout, or low-intensity chemotherapy—are considered more compatible with subsequent leukapheresis. Whenever possible, early leukapheresis prior to extensive salvage therapy may further optimize T-cell quality for CAR T-cell manufacturing.

An alternative strategy involves transducing marrow-infiltrating lymphocytes (MILs), which are polyclonal autologous T-cells derived from the bone marrow. MILs exhibit lower expression of exhaustion markers and greater stemness, polyfunctionality, and cytolytic activity in vitro [186,187].

A major hurdle in CAR T-cell therapy remains the long vein-to-vein time, typically spanning 14–21 days or more. Reducing manufacturing time is critical, particularly for patients with rapidly progressing disease. Notably, shorter manufacturing times have been associated with less differentiated T-cells. As a consequence, in vivo expansion is improved even at lower infusion doses, as previously reported [188,189]. Li et al. [190] reported encouraging results from a non-viral CAR T-cell manufacturing process completed within 3 days, achieving complete responses in three out of four patients using relatively low cell doses while maintaining expansion peaks comparable to approved products. Other examples include CD5-knockout CAR T-cells for nodal T-NHL produced in 5 days [191] and GLPG5101/GLPG5201 CAR T-cells, featuring early memory phenotypes and 7-day vein-to-vein times, currently under evaluation in R/R NHL and CLL [192,193]. Likewise, an anti-CD19 Fast-CAR-T therapy achieved MRD negativity in over 90% of B-ALL patients within one month, albeit with some high-grade CRS and ICANS events [194]. Finally, Stadel et al. [195] developed an ultra-fast manufacturing platform capable of producing CAR T-cells in under 24 h, with comparable CAR expression, strong expansion, and favorable preclinical efficacy. Their product, UF-Kure19, is now being evaluated in a phase I trial. Similar manufacturing times were also recently obtained with a novel platform starting from whole blood [196].

### 3.3. Allogeneic CAR T-Cells

As discussed before, conventional CAR T-cells require a complex manufacturing process in terms of timing and technical difficulties, leading to a significant risk of manufacturing failure. Allogeneic (allo) CAR T-cells could be a valuable off-the-shelf alternative that poses less logistic problems, as they are derived from a healthy donor instead of from a usually heavily pretreated patient. The main source of allogeneic lymphocytes for allo CAR T-cells is the pool of the peripheral blood mononuclear cells, while other possible sources include isolated stem cells from peripheral blood after CD34^+^ mobilization, umbilical cord blood, or induced pluripotent stem cells (iPSCs) [197].

The main obstacles of this approach are the risk of graft-versus-host disease (GVHD), since the TCR of the donor’s lymphocyte can recognize the healthy tissue of the patient, and the host-versus-graft phenomenon (HvG), where the patient’s immune system rejects the donor lymphocyte, thus hindering the allo CAR T-cells’ efficacy, expansion, and persistence. Different cellular sources offer variable degrees of HLA matching, potentially minimizing these complications [197].

Among the strategies to overcome allo CAR T-cell limitations, gene editing via different methods shows promising results. TCR-knockout-based approaches are widely used and have revealed GVHD rate reductions but not HvG risk mitigation [198]. Alternatively, elimination of both TCR- and MHC-class molecules can protect from both processes [199]. Other studies include CD47 overexpression as a way to reduce macrophage and NK activation towards allo CAR T-cells, CD52 knockout in combination with anti-CD52 mAb in order to suppress HvG and increase allo CAR T-cells persistence, and HLA-II molecule suppression via removal of the CIITA gene, protecting allo CAR T-cells form alloreactive T-cells of the host. Another approach for minimizing HLA-incompatibility is selecting specific T-cell populations that are less reliant on HLA recognition in their activity such as γδ T-cells, mucosal-associated invariant T (MAIT)-cells, cytokine-induced killer (CIK)-cells, invariant NK T-cells (iNKTs), and double-negative T-cells (DNTs) [200,201,202,203,204]. A notable example of this strategy is represented by CARCIK-CD19, an allogeneic CAR T-cell product engineered using the non-viral Sleeping Beauty transposon system. In a recent phase I/II clinical trial, CARCIK-CD19 demonstrated robust safety and efficacy in 36 patients with B-ALL relapsed after allogeneic hematopoietic stem cell transplantation. The product incorporated a CD28/OX40 third-generation CAR and showed high rates of durable CR and MRD negativity, with no cases of GVHD reported [205].

Compared to autologous CAR T-cell products, allo CAR T-cell development is lagging behind, with no products approved to date; nevertheless, several allo CAR T-cell products are being investigated. The most experience has been developed with anti-CD19 UCAR19/ALLO-501, in which gene editing was used to remove TCRα and CD52, thus adding in the lymphodepleting regimen the anti-CD52 mAb alemtuzumab. The product was evaluated in two multicenter studies, in which twenty-one B-ALL patients were treated, with only two cases of skin acute GVHD reported. Most patients received alemtuzumab, which enabled effective in vivo expansion. The global ORR was 67% (71% of which were MRD-negative), with a median duration of response (DOR) of 4.1 months. Two out of three patients receiving a second dose achieved a second CR [206]. Two multicenter phase I (ALPHA) and I/II (ALPHA2) studies were conducted on 33 LBCL patients. Lymphodepletion included ALLO-647, an anti-CD52 mAb. No GVHD or ICANS were reported. Globally, the CR rate was 58%, with a median duration of CR of 23.1 months and median PFS of 3.9 months (24 months in patients achieving CR) [207]. A similar product is being evaluated in B-ALL and acute myeloid leukemia (AML) patients targeting CD22 and CD123, respectively [208,209]. An interim analysis is available for a phase I trial investigating the anti-BCMA allo CAR T-cell ALLO-715 for R/R MM patients. The ORR was over 70%, with a mDOR of 8.3 months. A good safety profile in terms of CRS and neurotoxicity and no cases of GVHD were reported. [210]. Albeit with a different genome-editing technology, good responses were seen with the anti-CD19 allo CAR-T-cell PBCAR0191 (azercabtagene zapreleucel) following an enhanced lymphodepletion chemotherapy regimen in B-ALL and B-NHL. Among the twenty-one enrolled patients, the CR was 62% in B-NHL and 80% in B-ALL, with no GVHD reported, and there was only one grade 3 CRS [211]. A similar manufacturing process was used for PBCAR19B, a product able to evade immune rejection via suppression of HLA I and expression of HLA-E, which has recently been tested in R/R B-NHL with low toxicity rates and promising response rates [212]. CTX110 is an anti-CD19 allo CAR T-cell produced with the CRISPR/Cas9 technology that achieved good results in a phase I study conducted on B-NHL patients both in terms of toxicity and treatment response rates [213]. CB-010 is an anti-CD19 allo CAR T-cell which is being evaluated in the multicenter phase I ANTLER study in B-NHL and features PD1 knockout as a way to reduce cellular exhaustion [214]. Allo CAR T-cells produced with non-gene-editing technologies showed promising results both in R/R MM and B-NHL patients [215,216]. Finally, a good safety profile was also reported in the phase I trial for the iPSC-derived anti-CD19 allo CAR T-cell FT819 [217]. Of particular interest is allo CAR T-cell development for T-ALL, where autologous product development is hindered by the concern of transducing blast cells. Early data from trials testing anti-CD7 allo CAR T-cells edited with CRISPR/Cas9 in patients with R/R T-ALL have been promising, with CR rates up to 91% [218,219].

### 3.4. Other Effector Cells

#### 3.4.1. CAR NKs

As an alternative to T-cells, CAR NK-cells (CAR NKs) have been tested in various settings, including hematological malignancies. NK-cells belong to the innate immune system and offer many advantages over T-cells, such as reduced CRS and ICANS rates, tumor targeting without pre-sensitization or HLA-matching, and independence from major histocompatibility complex (MHC) recognition [13,220]. These characteristics grant an easier availability, tumor killing in case of MHC downregulation, and the possibility of using allogeneic sources without triggering GVHD.

The CAR structure and mechanism of transduction are similar to CAR T-cells, while the sources of NK-cells are multiple: banked NK-cell lines, peripheral blood mononuclear cells, umbilical cord blood, and iPSCs [221]. Despite these advantages, many challenges remain in developing an efficient CAR NK, including reduced expansion potential of the selected cell source, reduced cellular persistence, antigen expression and ability to mediate innate immune response, exhaustion, and sensibility to inhibitory signals mediated by the immune system and TME [222]. Similarly to CAR T-cells, CAR NKs able to release mediators such as IL15 or target multiple antigens are being developed and tested [223,224]. Liu et al. [225] conducted a phase I/II trial on B-NHL and CLL patients using HLA-mismatched, cord-blood-derived anti-CD19 CAR NKs, showing no CRS, ICANS, or GVHD and consistent response rates, especially in indolent diseases. Bachanova et al. [226] obtained similar promising safety and efficacy results utilizing an IPSC-derived CAR NK product. Another way to optimize CAR NKs’ efficacy in lymphoma is to combine them with monoclonal antibodies (mAbs) such as anti-CD20 and anti-CD79 or targeting different antigens such as CD22, potentially overcoming antigen loss [227].

CAR NKs have also been evaluated against MM. FT576 is an IPCS-derived CAR NK targeting BCMA with the coexpression of a recombinant IL-15 signaling complex (IL15RF) for autonomous persistence and a functionally enhanced high affinity. This construct showed interesting results in pre-clinical and phase I studies both in monotherapy and in combination with anti-CD38, anti-SLAMF7, and anti-CD19 monoclonal antibodies [228,229,230]. Other explored CAR NK targets in MM with preliminary encouraging activity include CS1 and GPRC5D [231,232,233]. Pre-clinical studies suggest that CAR NK efficacy against MM may be further enhanced by targeting multiple neoplastic receptors such as GPRC5D/CD38 or GPRC5D/BCMA or focusing on other targets such as CD70 [234,235].

#### 3.4.2. CAR Macrophages

CAR macrophages (CAR Ms) are a novel construct that can mediate an antitumor effect via antigen-dependent phagocytosis [236]. Among the advantages of CAR Ms compared to CAR T-cells are enhanced tumor infiltration, better immune cell trafficking, and reduced susceptibility to an immunosuppressive microenvironment with less tendency to exhaustion [237]. Beyond their cytotoxic and phagocytic roles, CAR Ms offer further unique advantages including the capacity to prime and activate T-cells through antigen spreading and to mitigate antigen escape mechanisms. [238].

The structure of CARs in engineered macrophages mirrors that of CAR T-cells, with the incorporation of a costimulatory domain enhancing their functional activity. CAR Ms not only exhibit phagocytic capabilities but also secrete a variety of mediators and cytokines, depending largely on their polarization phenotype, which can vary from pro-inflammatory M1—characterized by anti-tumoral activity—to M2, which is associated with immunoregulatory functions and generally considered pro-tumoral. One of the major challenges in the development of effective CAR M therapies lies in promoting and maintaining the M1 phenotype while resisting polarization toward the M2 state. Other critical factors include achieving robust cellular proliferation and persistence, challenges also encountered in other CAR-based therapies [239,240]. At present, no clinical trials have evaluated CAR Ms in hematologic malignancies, while the first in-human phase I trial with the anti-HER2 CAR-M product CT-0508 (NCT04660929) is ongoing in HER2-positive solid tumors.

Ongoing clinical trials evaluating novel CAR T-cell products in hematological malignancies are summarized in Table 1.

### 3.5. CAR T-Cells Plus X: Complementary Molecular Agents

#### 3.5.1. PD-1/PD-L1 Blockade

Blockade of the PD-1/PD-L1 axis may represent a viable strategy to mitigate TME-mediated effector T-cell exhaustion. In the phase I/II ZUMA-6 trial, axi-cel followed by the anti-PD-L1 atezolizumab was evaluated in R/R LBCL. The results of the phase I cohort showed a best ORR and CR of 75% and 46%, respectively, and the CAR T-cell expansion, as measured by area under the curve in the first 28 days, was over 2-fold higher than the median observed in the pivotal ZUMA-1 trial. The results of the phase II part of the trial are pending [241]. In a cohort of 14 patients with R/R B-NHL, the combination of CAR T-cells and the anti-PD1 mAb nivolumab resulted in a CR rate of 45.5%, with a median PFS of 6 months [242]. Moreover, nivolumab has been shown to enhance cytotoxicity and cytokine secretion in anti-CD19 CAR NKs [243]. In the phase Ib PORTIA trial, 12 patients received tisa-cel on day 0 and pembrolizumab every 21 days for up to six doses, initiating its administration on day -1 or after CAR T-cell infusion in different cohorts. The combination was safe and effective, and the cohort initiating pembrolizumab on day -1 appeared to show more favorable outcomes. Pembrolizumab did not lead to increased CAR T-cell expansion but delayed peak expansion if initiated on day -1 [244]. Preliminary data from the PLATFORM study describe 11 patients with B-NHL treated with liso-cel and receiving monthly infusions of durvalumab (an anti–PD-L1 mAb) for up to 12 cycles, achieving 7 CR [245]. In another clinical trial, the combination of anti-CD19 CAR T-cells with durvalumab was investigated in patients with R/R LBCL, administered for up to 10 monthly doses. Patients receiving durvalumab immediately before CAR T-cell infusion appeared to have lower response rates compared those treated after CAR T-cell infusion. Furthermore, retrospective comparison with a prior trial using the same CAR T-cell product without PD-L1 blockade (NCT01865617) yielded mixed results regarding the benefit of the combination therapy. Notably, patients receiving durvalumab after CAR T-cell infusion showed even lower ORR and CR rates compared to patients treated with CAR T-cells alone. Nevertheless, the DOR was longer among patients receiving combination therapy, suggesting that optimal timing of anti-PD-1/PD-L1 mAbs administration may be critical to enhancing CAR T-cell efficacy [246]. In a phase I/II clinical trial, 12 patients with B-NHL, who were either refractory to or relapsed after anti-CD19 CAR-T-cell therapy, received pembrolizumab every 21 days for up to 1 year. For the entire cohort the first pembrolizumab dose was administered at median of 3.3 months post-CAR T-cell infusion, and the median number of pembrolizumab doses was 2 (range 1–9). Interestingly, re-expansion of CAR T-cells in response to the first dose of pembrolizumab was observed in 10 patients, although ORR remained low. Of note is the fact that the authors reported that CAR T-cell expansion occurred in multiple waves in responding patients after pembrolizumab initiation, suggesting a correlation between expansion kinetics and response quality [247].

Pembrolizumab was also explored in combination with the anti-CD19/CD22 dual-targeting CAR T-cell AUTO3 In the ALEXANDER study. The ORR was 66.0%, including a CR rate of 48.9%. The median DOR was 8.3 months, with most patients in CR remaining relapse-free for over 12 months [248].

#### 3.5.2. Bruton Tyrosine Kinase Inhibitors

Bruton tyrosine kinase inhibitors (BTKis) may support CAR T-cell functionality by modulating the TME, the surface expression of target antigens, and cytokine secretion profiles. An in vitro study of cells derived from liso-cel demonstrated that prolonged exposure to two different BTKi compounds ibrutinib and acalabrutinib led to enhanced expansion and increased secretion of pro-inflammatory and immunostimulatory cytokines, without impairing cytotoxic T-cell function [249,250].

In patients with CLL, pre-leukapheresis exposure to ibrutinib improved the quality of collected cells by reducing surface expression of PD-1 and CD200. In murine models, post-infusion administration of ibrutinib enhanced CAR T-cell expansion and survival [249,251,252]. Encouraging results have emerged from the concurrent administration of CD19-directed CAR T-cells and ibrutinib in patients with R/R CLL. Compared to a matched cohort of 19 patients receiving CAR T-cell monotherapy, the combination group experienced lower rates of CRS, with comparable efficacy [253]. A phase II trial aimed to provide a more definitive therapeutic approach in CLL patients who had been on ibrutinib for at least 6 months without achieving CR. In this study, 19 patients continued BTKi therapy during CAR T-cell administration. Despite the 3-month CR rate being 44%, 72% of patients achieved undetectable MRD at 12 months [254]. In the phase II TARMAC study, 20 patients with R/R MCL received ibrutinib combined with tisa-cel starting 1 week prior to leukapheresis and continuing for a minimum of 6 months post-CAR T-cell infusion. The 4-month CR rate was 80%, and MRD negativity was achieved in 70% and 40% of patients tested by flow cytometry and molecular methods, respectively. Efficacy was consistent regardless of prior BTKi exposure or TP53 status, and longer ibrutinib exposure displayed lower levels of exhaustion markers and higher expansion peaks [255]. In a subset of patients relapsing after CAR T-cell and refractory to salvage ibrutinib (three FL and four MCL), a second infusion of anti-CD19 CAR T-cells combined with ibrutinib resulted in six CRs and one PR [256].

The use of BTKis as maintenance after CAR T-cell treatment has been investigated with encouraging results. In retrospective and prospective studies conducted in China, the use of BTKis (ibrutinib, Zanubrutinib, or orelabrutinib) following anti-CD19 CAR T-cells in R/R B-NHL resulted in prolonged treatment responses, with a potential benefit compared to CAR T-cell monotherapy. While peak CAR T-cell expansion was not markedly affected by BTKis, co-treatment was associated with improved persistence and reduced exhaustion [257,258,259].

A retrospective study included 54 patients with R/R LBCL who received response-adapted zanubrutinib plus tislelizumab (anti–PD-1 mAb) after CAR T-cells. With a median follow-up of almost 2 years, the 6-month ORR was 80% (CR 76%), and median PFS and OS were not reached [260].

#### 3.5.3. Immunomodulatory Agents

Lenalidomide has demonstrated the ability to enhance the therapeutic efficacy of anti-CS1 or anti-BCAM, anti-CD19, anti-CD23, and anti-WT1 CAR T-cells in preclinical models of MM, DLBCL, CLL, and AML, respectively. This enhancement occurs through several mechanisms, including the dose-dependent preferential expansion of CD8^+^ T-cells, increased production of proinflammatory cytokines, promotion of Th1 polarization, extended persistence of CAR T-cells, and overall improved effector functions [261,262,263,264,265,266]. In a clinical setting of MM patients, the safety and efficacy of a sequential treatment regimen consisting of autologous stem cell transplantation followed by anti-CD19 and anti-BCMA CAR T-cell infusion plus lenalidomide maintenance until disease progression was investigated. Seven out of ten patients maintained MRD negativity for over 2 years, and neither median OS nor median PFS were reached with a median follow-up of 42 months. Only low grades of CRS occurred, and no ICANS events were reported [267]. In another study, Garfall et al. [268] enrolled patients with MM and low tumor burden across two cohorts. Patients responding to first-line treatment were allocated to receive either anti-BCMA CAR T-cells or a sequential combination of anti-BCMA and anti-CD19 CAR T-cells. All subgroups received maintenance therapy with either pomalidomide or lenalidomide. Interestingly, the kinetics of CAR T-cell re-expansion appeared temporally associated with the initiation of maintenance therapy, suggesting a delayed in vivo reactivation of CAR T-cells that was seemingly independent of their initial expansion.

#### 3.5.4. BCR/ABL Inhibitors

Among the approved BCR/ABL inhibitors, dasatinib exerts multiple effects on the TME. It binds to and inhibits SRC family kinases, thereby preventing T-cell activation following antigen encounter and modulating the T-cell epigenome. This mechanism may serve a dual purpose: mitigating CAR T-cell-therapy-related toxicities and preventing functional exhaustion of CAR T-cells, resulting in a reversible dose- and time-dependent adjuvant effect [269,270,271]. In a non-randomized phase II trial enrolling 28 adults with Philadelphia-chromosome-positive (Ph+) B-ALL, dasatinib was administered as part of the induction regimen, followed by sequential CD19 and CD22 CAR T-cells, and this was continued as maintenance. Among 27 evaluable patients, 25 responses were observed (21 complete molecular responses) and the 2 y OS and leukemia-free survival rates were over 90%. Only grade 1 CRS events and no neurological toxicities were observed [272].

#### 3.5.5. Bcl-2 Inhibitors

In malignant B-cell cultures, including B-ALL and lymphoma lines, pre-treatment of CD19-directed CAR T-cells with Bcl-2 inhibitors such as venetoclax resulted in enhanced cellular longevity, increased cytokine secretion, and improved cytotoxic function. This was accompanied by higher surface expression of CD19 and increased levels of the pro-apoptotic protein BAK. However, concurrent or post-infusion administration of Bcl-2 inhibitors elicited adverse effects on CAR T-cells, including reduced expansion, functional exhaustion, and long-term cytotoxic impairment [273]

#### 3.5.6. γ-Secretase Inhibitors

BCMA is cleaved by γ-secretase, reducing its surface expression and increasing soluble BCMA (sBCMA), which may act as a decoy receptor for CAR T-cells. Inhibition of γ-secretase enhances BCMA surface density and CAR T-cell efficacy in vitro, though high doses of γ-secretase inhibitors (GSIs) can impair CAR T-cell expansion [274]. In a first in-human trial, the GSI crenigacestat combined with BCMA CAR T-cells increased surface BCMA and reduced sBCMA, with a median PFS of 11 months. Stratification showed significant differences between anti-BCMA-naïve and previously anti-BCMA-exposed patients (median PFS was 28.8 and 2.6 months, respectively). No correlation was found between CAR T-cell dose and clinical outcomes, suggesting antigen density may play a compensatory role. However, ICANS occurred in 38% of patients [275]. Single-cell analysis revealed that prior exposure to BCMA-targeted therapies attenuated the BCMA upregulation induced by GSI [276].

#### 3.5.7. Other Approaches

Several other compounds have been investigated in preclinical and phase I studies, showing promising results in enhancing CAR T-cell activity through various mechanisms, including upregulation of death ligands and stress-related proteins; downregulation of class I HLA molecules and increased expression of target antigens on neoplastic cells; modulation of gene expression promoting naïve and memory T-cell differentiation; improved cytotoxic and secretory functions; reduced CAR T-cell exhaustion; enhanced recruitment of anti-CD19 CAR T-cells; and promotion of a CD8^+^ phenotype. These treatment categories include proteasome inhibitors, hypomethylating agents, EZH2 inhibitors, FLT3 inhibitors, and all-trans retinoic acid [277,278,279,280,281,282,283,284,285,286,287,288]. In addition, novel emerging agents are under investigation in order to further empower CAR T-cell efficacy [76,289,290,291,292,293]

CD20xCD3 bispecific mAbs have demonstrated high efficacy in the treatment of R/R B-NHLs and recently received approval in this setting [294,295,296,297]. Based on their encouraging activity, phase I–II trials are currently underway evaluating their combination with CAR T-cell therapy, with the aim of further improving cure rates in high-risk patients.

A summary of the large array of clinical trials currently underway to evaluate novel combinations aimed at overcoming resistance to CAR T-cell therapy is provided in Table 2.

## 4. Discussion

CAR T-cell therapy has profoundly reshaped the treatment of hematologic malignancies, yet resistance and relapse remain frequent and multifactorial. Antigen escape is among the most common mechanisms and has prompted the development of dual-target CARs, such as anti-CD19/CD22 or anti-CD19/CD20, designed to reduce the risk of relapse due to single-antigen loss. Beyond target selection, low-affinity CARs have been engineered to attenuate tonic signaling and delay exhaustion, thereby improving persistence. Other next-generation platforms, including CAR TRUCKs and armored CARs, aim to remodel the tumor microenvironment by secreting cytokines or providing additional costimulatory signals.

T-cell intrinsic quality is equally important. Early harvesting, performed before multiple cytotoxic therapies, preserves naïve and stem-like memory subsets, improving both manufacturing success and long-term persistence. Better T-cell selection and the exploration of novel fast manufacturing platforms are also being investigated to optimize the starting material and product composition. In parallel, the development of allogeneic CAR T-cells derived from healthy donors offers a potential solution to the limitations of autologous approaches, providing more rapid and standardized availability, though issues of rejection and long-term safety remain to be addressed.

The tumor microenvironment represents a further critical barrier, imposing metabolic restrictions, fostering suppressive immune populations, and upregulating inhibitory pathways. In this setting, combining CAR T-cells with immune checkpoint inhibitors—particularly PD-1/PD-L1 blockade—appears one of the most clinically translatable strategies, supported by extensive oncologic experience. Additional “CAR T plus X” combinations, including BTK inhibitors, immunomodulatory drugs, and other small molecules, have also shown encouraging synergistic effects, enhancing expansion, activity, and persistence through complementary mechanisms. Finally, host-related factors such as the intestinal microbiota have been associated with CAR T-cell expansion, persistence, and toxicity, although their clinical application remains largely exploratory compared with other, more directly applicable strategies.

## 5. Conclusions

Resistance to CAR T-cell therapy arises from tumor-intrinsic alterations, intrinsic properties of the CAR T-cell product, and extrinsic host- and microenvironment-related influences. To overcome these challenges, multiple complementary strategies are being developed. Dual-target constructs reduce the likelihood of antigen escape, while low-affinity CARs can mitigate exhaustion and improve persistence. CAR TRUCKs and armored CARs represent rational approaches to remodel the tumor milieu and sustain function under suppressive conditions. Early harvesting and improved T-cell selection enhance the quality of the autologous starting material, and novel manufacturing platforms may further refine product composition. Allogeneic CARs may eventually provide off-the-shelf solutions, potentially shortening treatment delays and standardizing availability. Combination strategies—particularly with checkpoint inhibitors, but also with BTK inhibitors, immunomodulatory drugs, and other agents—hold promise for enhancing persistence and durability of response. Even host-related modulators, such as the microbiome, have been implicated in influencing CAR T-cell efficacy, though their role is still largely exploratory.

Altogether, these approaches highlight the multifaceted nature of resistance and the need for integrative solutions that simultaneously address antigen escape, product quality, and extrinsic barriers, thereby progressively moving the field toward more effective and accessible therapies.

## 6. Future Directions

Looking forward, the evolution of CAR T-cell therapy is likely to progress along two complementary tracks. On the one hand, futuristic innovations such as logic-gated CARs, advanced armored constructs, and genome-edited platforms promise to enhance specificity, persistence, and resistance to immunosuppression, although their broad implementation in clinical practice will require further validation and long-term follow-up. On the other hand, several strategies appear readily translatable to current care. The combination of CAR T-cells with checkpoint inhibitors or other small molecules, the implementation of dual-target constructs, the design of low-affinity CARs, and the adoption of early harvesting approaches represent interventions that can feasibly be integrated into practice in the near future.

The panorama of CAR T-cell therapy is rapidly evolving, and the best is yet to come.

## Figures and Tables

**Table 1:** Recent phase 1–2 clinical trials involving novel CAR products in hematological malignancies.

NCT Number Ref. (See Below)	Intervention	Disease
1.	CT1190B CAR-T	R/R B-NHL
2.	IL-6-silenced CD19 CAR-T	R/R B-cell lymphoma
3.	Rapid production CD19 CAR-T	R/R B-ALL and B-NHL
4.	CD19 CAR-NK	R/R B-ALL
5.	CD19 CAR-T secreting IL18	R/R B-ALL
6.	CD19 CAR-T with TLR2	R/R B-cell lymphoma
7.	CD19 CAR-NK	R/R CNS lymphoma
8.	CD19 CAR-T/CAR-NK	R/R B-cell malignancies
9.	CD19/22 CAR-T	R/R B-ALL
10.	CD19/22 CAR-T	R/R pediatric B-ALL
11.	Sequential CD19 and CD22 allogeneic CAR-T	R/R B-ALL
12.	Metabolically armored CD19 CAR-T	R/R B-cell malignancies
13.	Persistence-enhanced CD19 CAR-T	R/R B-NHL
14.	BCOR and ZC3H12 KO CD19 CAR-T	R/R B-cell lymphoma and B-ALL
15.	CD20 CAR-T	R/R B-NHL
16.	Sequential CD19 CAR-NK and CD7/19 CAR-T	B-NHL
17.	CD19/22 CAR-T	R/R B-cell leukemia and lymphoma
18.	CD19/CD22 TLR2 CAR-T	R/R B-ALL and B-NHL
19.	DuoCAR20.19.22-D95	R/R B-Cell malignancies
20.	C402-CD19-CAR	R/R B-NHL
21.	CIK cell therapy	R/R B-ALL
22.	CD19/CD20 CAR-T	R/R B-cell malignancies
23.	CD19/TGF-beta CAR-T	R/R LBCL
24.	CD19/79b CAR-T	R/R B-NHL
25.	CD19/IL10 CB CAR-NK	R/R B-NHL
26.	CD19/BAFF CAR-T	R/R B-cell malignancies
27.	CD22 CAR-T	R/R B-cell leukemia and lymphoma
28.	CD20/CD30 CAR-T	R/R lymphomas
29.	CD5 CAR-T	R/R T-cell leukemia and lymphoma
30.	CD5 KO CD5 CAR-T	R/R T-NHL
31.	CD5 CAR-NK	R/R T-ALL or T-cell lymphoma
32.	CD5 CAR-NK secreting IL15	R/R NK/T-cell malignancies
33.	Sequential CD5/7 CAR-T	R/R T-cell leukemia and lymphoma
34.	CD7 CAR-T	R/R T-ALL or T-cell lymphoma
35.	CD7 CAR-T	R/R NK/T-cell malignancies
36.	CD7 CAR NK	R/R T-ALL
37.	Autologous and allogeneic CD7 CAR-T	R/R T-cell leukemia and lymphoma
38.	Dual-epitope BCMA CAR-T	R/R MM
39.	BCMA CAR-NK	R/R MM and PCL
40.	BCMA/CD19 CAR-T	R/R aggressive B-NHL
41.	BCMA/CD19 CAR-T	R/R MM
42.	BCMA/CD19 CAR-T	R/R MM, B-ALL, and B-NHL
43.	BCMA/GPCR5D CAR-T (RD140)	R/R MM and PCL
44.	BCMA/GPCR5D CAR-T	R/R MM
45.	BCMA/TGF-beta CAR-T	R/R MM
46.	BCMA/FcRL5 CAR-T	R/R MM
47.	CD19/22/BCMA CAR-T	R/R B-NHL
48.	LCAR-M61S and LCAR-M61D	R/R MM
49.	CD27 armored BCMA CAR-T	R/R MM
50.	UF-KURE-BCMA CAR-T	R/R MM
51.	GPRC5D CAR-T	R/R MM
52.	GPRC5D/CD19 CAR-T	R/R MM
53.	CD30 CAR-T	R/R CD30+ lymphoma
54.	CD70 CAR-T	R/R hematological malignancies
55.	CD70 CAR-NK	T-cell leukemia and lymphoma
56.	CD38/CS1 CAR-T	R/R MM
57.	iC9/CAR19/IL15 CB CAR-NK	High-risk lymphoma patients with primary Sjogren’s syndrome
58.	CD19/CD22/BCMA CAR-T	R/R MM
59.	FcR L5 CAR-T	R/R MM
60.	BAFFR-based CAR-T	R/R B-NHL
61.	CS1 CAR-T	R/R MM
62.	IL7R-modified CD30 CAR-T	R/R CD30^+^ lymphoma
63.	APRIL-BAFF-Bicephali CAR-T	R/R MM
64.	EGFRt/19-28z/IL-12 CAR T	R/R CD19^+^ malignancies
65.	NKG2 CAR-NK	R/R MM
66.	BAFF CAR-T	R/R NHL
67.	CAR-T co-expressing IL15	R/R hematological malignancies
68.	BAFF CAR-T	R/R MM
69.	Allogeneic CD19 CAR-T	R/R B-ALL and B-NHL
70.	Allogeneic CD20 CAR-T	R/R B-NHL
71.	Allogeneic CD19/20 CAR-T	R/R B-cell malignancies
72.	Allogeneic CD19/22 CAR-T	R/R B-ALL and B-NHL
73.	Allogeneic BCMA CAR-T	R/R MM
74.	Allogeneic CD19/BCMA CAR-T	R/R B-cell malignancies
75.	Allogeneic BCMA/GPCR5D CAR-T	R/R MM
76.	Allogeneic CAR-T (CT0596)	PCL
77.	TRAC and SPPL3 KO allogeneic CD19 CAR-T	R/R B-NHL
78.	Allogeneic BCMA or CD138 or CD38 or CD19 CAR-T	R/R MM
79.	TmCD19-IL18 CAR T	R/R CD19^+^ malignancies

Abbreviations: ALL (acute lymphoblastic leukemia), BAFF (B-cell activating factor), BAFFR (B-cell activating factor receptor), BCMA (B-cell maturation antigen), CAR (chimeric antigen receptor), CIK (cytokine-induced killer cells), CNS (central nervous system), FcRL5 (Fc Receptor-Like 5), GPRC5D (G-protein coupled receptor family C group 5 member D), IL (interleukin), KO (knockout), MM (multiple myeloma), NCT (national clinical trial), NHL (non-Hodgkin’s lymphoma), NK (natural killer), PCL (plasma cell leukemia), ref. (reference), R/R (relapsed or refractory), SPPL3 (Signal Peptide Peptidase-Like 3), TGF (tumor growth factor), TLR2 (toll-like receptor 2), TRAC (T-cell receptor alpha constant). NCT references: (1) NCT07032324; (2) NCT06987916; (3) NCT05779930, NCT06561425; (4) NCT06631040; (5) NCT06287528; (6) NCT06486051; (7) NCT06827782; (8) NCT06596057; (9) NCT06927466; (10) NCT06752785, NCT06777979; (11) NCT06326008; (12) NCT06716164, NCT06393335; (13) NCT06544265; (14) NCT07009002, NCT07008885; (15) NCT06364852, NCT06326463, NCT06002659, NCT06248086, NCT06539338; (16) NCT06464861; (17) NCT06735495, NCT06445803, NCT06213636, NCT06081478, NCT06078306, NCT06880913, NCT06834529, NCT06559189, NCT06559189; (18) NCT06879262; (19) NCT06879340; (20) NCT06830031; (21) NCT06389305; (22) NCT07024147, NCT06703892, NCT06508931, NCT06503094, NCT06395870, NCT06295549; (23) NCT06047197; (24) NCT06026319; (25) NCT06707259; (26) NCT06346912; (27) NCT06340737, NCT06285422, NCT06208735; (28) NCT06532643, NCT06519344; (29) NCT06874946, NCT06633341, NCT06633354, NCT06316856, NCT07022964; (30) NCT06420089; (31) NCT06909474, NCT06514794; (32) NCT06699771; (33) NCT06420076; (34) NCT06934382, NCT06136364, NCT07008872, NCT06064903, NCT05979792, NCT06925464; (35) NCT06732492; (36) NCT06849401; (37) NCT06316427; (38) NCT06503107; (39) NCT06045091; (40) NCT06097455; (41) NCT07003555, NCT06235229, NCT06235229, NCT06986434; (42) NCT06961669; (43) NCT06655519; (44) NCT06644443, NCT06515262, NCT07003568, NCT06644118, NCT06153251, NCT05998928; (45) NCT05976555; (46) NCT06759181; (47) NCT06446128; (48) NCT06472479; (49) NCT06705725; (50) NCT06698744; (51) NCT06407947, NCT06333509, NCT06297226, NCT06271252, NCT06084962, NCT06615479; (52) NCT06298266; (53) NCT06850285; (54) NCT06492304, NCT06345027, NCT05948033; (55) NCT06696846, NCT06633341; (56) NCT06574958; (57) NCT06967038; (58) NCT06732232; (59) NCT06196255; (60) NCT06191887; (61) NCT06185751; (62) NCT06176690; (63) NCT06132711; (64) NCT06343376; (65) NCT06379451; (66) NCT06916767; (67) NCT06783816; (68) NCT05546723; (69) NCT06481735, NCT06323525, NCT06314828, NCT06304636, NCT06256484, NCT06080191, NCT04881240, NCT06503211, NCT06838832, NCT06793241, NCT06696833, NCT06662227; (70) NCT06313957; (71) NCT06014762; (72) NCT06009107, NCT06005649; (73) NCT06663046; (74) NCT06976437; (75) NCT06594211; (76) NCT06988059, NCT06730256; (77) NCT06014073; (78) NCT06006741; (79) NCT05989204.

**Table 2:** Ongoing phase 1–3 clinical trials evaluating CAR T-cells plus X combinations in hematological malignancies.

NCT Number	Intervention	Disease
NCT05310591	CD19 CAR-T + Nivolumab	B-ALL
NCT05385263	CD19 CAR-T + Nivolumab	DLBCL
NCT04205409	(Post CAR-T) Nivolumab	R/R B-NHL, R/R MM
NCT05352828	CD30 CAR-T + Nivolumab	R/R cHL
NCT04134325	CD30 CAR-T + Nivolumab OR Pembrolizumab	R/R cHL
NCT06767956	(Post CD19 CAR-T) Nivolumab + Golcadomide,	R/R B-NHL
NCT06523621	(Post idecabtagene Vicleucel) Nivolumab	R/R MM
NCT05934448	CAR-T + Pembrolizumab	R/R PMBCL
NCT06242834	(Post CAR-T/ASCT) Pembrolizumab + Tazemetostat	R/R B-NHL
NCT05659628	CD19 CAR-T + Tislelizumab	R/R DLBCL
NCT06876688	Relmacabtagene autoleucel + Tislelizumab ± BTKi	R/R PCNSL
NCT04539444 (Uknown status)	CD19/22 CAR-T + Tislelizumab	R/R B-NHL
NCT00586391	CD19 CAR-T + Ipilimumab	R/R B-NHL R/R ALL, R/R CLL
NCT03331198	Lisocabtagene maraleucel + Ibrutinib or Venetoclax	R/R CLL/SLL
NCT03960840	Rapcabtagene autoleucel + Ibrutinib	R/R CLL/SLL
NCT06482684	Brexucabtagene autoleucel + Ibrutinib	MCL
NCT04234061	Tisagenlecleucel + Ibrutinib	R/R MCL
NCT05672173	Lisocabtagene maraleucel + Ibrutinib + Nivolumab	Richter’s Syndrome
NCT05744037 (Uknown status)	CD19 CAR-T + Ibrutinib	R/R B-NHL
NCT05202782	CAR-T + Zanubrutinib	R/R B-NHL
NCT05873712	Lisocabtagene maraleucel + Zanubrutinib	Richter’s Syndrome
NCT06646666	CAR-T + ATRA + Zanubrutinib ± radiotherapy ± PD-1 inhibitor	R/R B-NHL
NCT06695013	Zanubrutinib ± radiotherapy + CAR-T ± Zanubrutinib and Tislelizumab	R/R B-NHL
NCT05871684	CAR-T + Zanubrutinib + Tislelizumab	R/R B-NHL
NCT06167785	(Post CD19 CAR-T) Zanubrutinib + Tislelizumab	R/R B-NHL
NCT05020392	CD19 CAR-T + Zanubrutinib/Ibrutinib/Orelabrutinib	R/R B-NHL
NCT05495464	Acalabrutinib + Rituximab + Brexucabtagene autoleucel	MCL
NCT05256641	CD19 CAR-T + Acalabrutinib	R/R B-NHL
NCT04257578	Axicabtagene ciloleucel + Acalabrutinib	R/R B-NHL
NCT04484012	CD19 CAR-T + Acalabrutinib	R/R MCL
NCT05990465	CD19 CAR-T + Pirtobrutinib	R/R B-NHL
NCT06553872	Brexucabtagene autoleucel + Pirtobrutinib	R/R MCL
NCT06553872	CD19 CAR-T Brexucabtagene autoleucel + Pirtobrutinib	MZL
NCT06336395	CAR-T If high risk: + Imatinib/Dasatinib	B-ALL Ph+
NCT05523661	CD19/CD22 CAR-T + Dasatinib	ALL Ph+
NCT05993949	Brexucabtagene autoleucel + Dasatinib	R/R B-ALL
NCT04603872	CD19/BCMA CAR-T + Dasatinib	R/R ALL, R/R B-NHL, R/R MM
NCT06940297	Ciltacabtagene Autoleucel + Dasatinib + Quercetin	R/R MM
NCT05934838	CAR-T + Tazemetostat	R/R B-NHL
NCT06793475	BCMA/GPRC5D CAR-T + Thalidomide+ Apornemin	R/R MM
NCT03070327	BCMA CAR-T + Lenalidomide	MM
NCT05840107	BCMA/CD19 CAR-T + Lenalidomide	MM
NCT06913192	ASCT + BCMA CAR-T + Lenalidomide ± Bortezomib	MM
NCT04196491	Idecabtagene vicleucel + Lenalidomide	MM
NCT06762431	CD19 CAR-T + Lenalidomide	R/R CLL
NCT04935580 (Uknown status)	BCMA/CD19 CAR-T + Lenalidomide	R/R MM
NCT05860036	BCMA CAR-T Consolidation: Lenalidomide + Bortezomib Maintenance: Lenalidomide	MM
NCT05850286	BCMA CAR-T + Consolidation + ASCT + BCMA CAR-T + Lenalidomide	MM
NCT07045909	Anitocabtagene Autoleucel + Lenalidomide	MM
NCT04133636	Ciltacabtagene autoleucel + Lenalidomide	MM
NCT03601078	Idecabtagene vicleucel + Lenalidomide	MM
NCT05257083	Ciltacabtagene autoleucel + Lenalidomide vs. SoC	MM
NCT06045806	(Post ASCT) Idecabtagene vicleucel + Lenalidomide	MM
NCT05870917	Induction: Lenalidomide + Bortezomib + first infusion of BCMA CAR-T Consolidation: Lenalidomide + Bortezomib + ASCT + second infusion of BCMA CAR-T Maintenance: Lenalidomide	PCL
NCT05979363	Induction: Lenalidomide + Bortezomib + BCMA CAR-T Consolidation: Lenalidomide + Bortezomib Maintenance: Lenalidomide + Bortezomib	PCL
NCT06414148	(MRD+ post CD19 CAR-T) Epcoritamab or Lenalidomide + Epcoritamab + Rituximab	R/R LBCL
NCT06179888	(Post-idecabtagene vicleucel) Iberdomide	R/R MM
NCT06121843	Arlocabtagene Autoleucel + Alnuctamab or Mezigdomide or Iberdomide	R/R MM
NCT06048250	Idecabtagene vicleucel + Mezigdomide	R/R MM
NCT06209619	CD19 CAR-T + Golcadomide + Rituximab	R/R B-NHL
NCT06271057	CD19 CAR-T + Golcadomide	R/R LBCL
NCT04850560 (Uknown status)	CD19 CAR-T + Decitabine	R/R B-NHL
NCT04337606	(Post CAR-T) Decitabine + Chidamide or Decitabine + Camrelizumab	R/R B-NHL
NCT04553393 (Uknown status)	Decitabine-primed CD19/CD20 CAR-T ± Chidamide or Decitabine or Chidamide + Decitabine	R/R B-NHL
NCT04093596	Anti-BCMA Allogeneic CAR-T ± Nirogacestat	R/R MM
NCT06464185	CD19 CAR-T + Glofitamab	R/R B-NHL
NCT06567366	CAR-T + Glofitamab	R/R LBCL
NCT04703686	(Post CD19 CAR-T) Obinutuzumab + Glofitamab	R/R B-NHL
NCT07003295	(Post CD19 CAR-T) Obinutuzumab + Glofitamab	R/R MCL
NCT06552572	(PR post CD19 CAR-T): Obinutuzumab + Glofitamab	R/R DLBCL
NCT06071871	(Post CAR-T): Obinutuzumab + Glofitamab + Polatuzumab vedotin	R/R LBCL
NCT06015880	(Post CAR-T): Mosunetuzumab + Polatuzumab vedotin + Lenalidomide	R/R B-NHL
NCT04889716	(Post CD19 CAR-T): Mosunetuzumab or Obinutuzumab + Glofitamab	R/R LBCL
NCT05260957	CAR-T + Mosunetuzumab + Polatuzumab vedotin	R/R B-NHL
NCT05633615	CD19 CAR-T + Mosunetuzumab or Polatuzumab vedotin or Mosunetuzumab + Polatuzumab vedotin	R/R B-NHL

Abbreviations: ALL (acute lymphoblastic leukemia), ASCT (autologous stem cell transplant), ATRA (all-trans retinoic acid), B-ALL (B-cell acute lymphoblastic leukemia), B-NHL (B-cell non-Hodgkin’s lymphoma), BCMA (B-cell maturation antigen), CAR-T (chimeric antigen receptor T-cell), CD (cluster of differentiation), cHL (classical Hodgkin’s lymphoma), CLL (chronic lymphocytic leukemia), CR (complete response), DLBCL (diffuse large B-cell lymphoma) LBCL (large B-cell lymphoma), MCL (mantle cell lymphoma), MM (multiple myeloma), MRD (minimal residual disease), MZL (marginal zone lymphoma), NCT (national clinical trial), PCL (plasma cell leukemia), PCNSL (primary central nervous system lymphoma), PD-1 (programmed cell death protein 1), PMBCL (primary mediastinal B-cell lymphoma), PR (partial response), R/R (relapsed or refractory), SLL (small lymphocytic lymphoma).
